# Prenatal diagnosis of Tay-Sachs disease: our institutional experience

**DOI:** 10.1186/1755-8166-7-S1-P124

**Published:** 2014-01-21

**Authors:** Jayesh Sheth, Mehul Mistri, Frenny Sheth, Sarita Gupta

**Affiliations:** 1 FRIGE’s Institute of Human Genetics, FRIGE House, Jodhpur Gam road, Satellite, Ahmedabad-380015, Gujarat, India; 2 M.S.University, Vadodara, India

## Introduction

Tay-Sachs disease (TSD) is the second most common storage disease after Guacher disease in the group of lipid storage disorders in India. In absence of any therapeutic option, prenatal diagnosis is the only way to prevent the disease burden.

## Aims and objectives

The present investigation was undertaken to provide cost effective prenatal diagnosis of TSD by enzyme based study from cultured chorionic trophoblast (CT)/uncultured CV or cultured amniotic fluid cells (AF) and further confirmation by molecular analysis of *HEXA* gene.

## Material and methods

Prenatal diagnosis was carried out in six women having confirmed index case with TSD was enrolled in this study. Enzyme study and molecular analysis was carried from CT in 3 women at 11-13 weeks of gestational age while in remaining 3 women cultured AF cells were used at 16 weeks of gestation. The β-Hexosaminidase-A activity was carried out with synthetic substrates, 4-methylumbelliferyl-6-sulfo-N-acetyl-beta-glucosaminide (4-MUGS) and DNA-based analysis was carried out by the bidirectional sequencing of *HEXA* gene. Written informed consent was obtained from all patients.

## Results and discussion

Deficiency of β-Hexosaminidase-A enzyme activity [21.7 nmol/hr/mg protein (NR: 271–1621 nmol/hr/mg protein)] from CT cells was detected in one case and mutation study observed homozygous mutation of 4bp insertion at c.1277_1278insTATC in the CT same as index case. Whereas, remaining 2 enzymatically normal fetuses were normal for earlier identified index case mutations [Homozygous mutation *D322N* and compound heterozygous mutations (D322N/c.1277_1278insTATC) respectively]. The enzyme activity was carried out from cultured AF cells in three women and deficiency of β-Hexosaminidase-A enzyme activity of 23.4 nmol/hr/mg protein (NR:271-2213.2 nmol/hr/mg protein) with homozygous mutations E114K in one case; intermediate enzyme activity of 247.2 nmol/hr/mg protein (~50% of mean) having heterozygous mutation *D322N* in one case and normal enzyme activity (670.5 nmol/hr/mg protein) was detected in one subject also showed absence of *W485X* mutation which was present in the index case.

## Conclusion

This study clearly demonstrates that for storage disorders like Tay-Sachs enzyme analysis from cultured/ uncultured CV and cultured AF provide highly reliable information for prenatal study and can be used as with high sensitivity and specificity for prenatal diagnosis of the disease where index case has confirmed diagnosis proven by enzyme activity or by molecular analysis.

